# Comprehensive biophysical and functional study of ziv-aflibercept: characterization and forced degradation

**DOI:** 10.1038/s41598-020-59465-7

**Published:** 2020-02-14

**Authors:** Jesús Hermosilla, Raquel Pérez-Robles, Antonio Salmerón-García, Salvador Casares, Jose Cabeza, Jonathan Bones, Natalia Navas

**Affiliations:** 1 Department of Analytical Chemistry/Institute for Biomedical Research (ibs.GRANADA), University of Granada, E-18071 Granada, Spain; 2 Department of Clinical Pharmacy, San Cecilio University Hospital, Institute for Biomedical Research, (ibs.GRANADA), 18016 Granada, Spain; 3 Department of Physical Chemistry/Institute of Biotechnology, University of Granada, E-18071 Granada, Spain; 4 Characterisation and Comparability Laboratory, NIBRT., Fosters Avenue, Mount Merrion, Blackrock, Co., Dublin, Ireland

**Keywords:** Health care, Therapeutics

## Abstract

Aflibercept (AFL) is an Fc fusion protein used in the treatment of colorectal cancers and different ophthalmological diseases. There are two medicines in which AFL is the active substance: Zaltrap and Eylea, referred as ziv-AFL and AFL respectively. No proper accelerated degradation studies were published on either AFL or ziv-AFL. These studies are essential during research, development and manufacturing stages. Here, we characterized ziv-AFL and submitted it to different stress conditions: light, 60 °C, freeze-thaw cycles, changes in pH, high hypertonic solution and strong denaturing conditions. We used an array of techniques to detect aggregation (SE-HPLC/DAD and DLS), changes in secondary structure (Far-UV circular dichroism), changes in conformation or tertiary structure (Intrinsic tryptophan fluorescence) and alterations in functionality (ELISA). Results indicate that aggregation is common degradation pathway. Two different types of aggregates were detected: dimers and high molecular weight aggregates attributed to β-amyloid-like structures. Secondary structure was maintained in most of the stress tests, while conformation was altered by almost all the tests except for the freeze-thaw cycles. Functionality, evaluated by its immunochemical reaction with VEGF, was found to be stable but with decrease when exposed to light and with likely partial inactivation of the drug when pH was altered.

## Introduction

Fc-fusions proteins are a well-established class of biotherapeutics. They are composed of the Fc region of the IgG antibody (Hinge-CH2-CH3) and a desired linked protein. Although the top-selling biotherapeutics are monoclonal antibodies (mAbs), there is increasing use of therapeutic Fc-fusion proteins in medicine today^1^. One of these is aflibercept (AFL), a recombinant human Fc-fusion protein that –as indicated in the assessment report of Zaltrap of the European Medicine Agency^2^– “acts as a high-affinity soluble decoy receptor that preferentially binds to vascular endothelial growth factor A (VEGF-A), VEGF-B and placenta growth factor (PlGF) and prevents these factors from activating their endogenous receptors. By blocking this pathway, AFL exerts direct anti-cancer activity and boosts the anti-cancer activity of chemotherapy agents by preventing new tumour vessel growth, regressing existing tumour vessels, normalizing vasculature, negatively affecting tumour cell function, offsetting the effects of chemotherapy induction of VEGF levels and potentially inhibiting VEGF repression of dendritic cell function”^2^. As a VEGF inhibitor, AFL is also classified as a VEGF-trap together with bevacizumab and ranibizumab.

Cancer therapy is not the only field of medicine to benefit from AFL, which is also being used to treat several ophthalmological diseases characterized by neovascularization. As the administration to patient varies depending on the particular illness they are suffering, two different medicines are currently available, Zaltrap (Sanofi-Aventis US, LLC, Bridgewater, New Jersey, USA and Regeneron Pharmaceuticals, Inc, Tarrytown, New York, USA), which is indicated for the treatment of metastatic colorectal carcinoma resistant to an oxaliplatin-containing regimen^3^ and Eylea (Regeneron, Tarrytown, New York, USA and Bayer Healthcare, Leverkusen, Germany), an ophthalmologic medicine approved for the treatment of wet age-related macular degeneration^4,5^ and macular oedema from retinal vein occlusion or diabetes^6^. AFL is identical in both medicines but differently formulated. In order to distinguish between the two, the AFL from Zaltrap is normally referred to as ziv-AFL.

Given the widespread, generalized incidence of the diseases they are approved for, these two medicines are used worldwide. From a commercial point of view, although both medicines are expensive, ziv-AFL is much cheaper than AFL^7^ and this has led clinical studies to be conducted to evaluate the possible use of ziv-AFL in the treatment of ocular diseases^7,8^. Surprisingly, only one of these studies evaluated the stability of ziv-AFL in *ad-hoc* formulations for intravitreal injection administration, although it only tested its capacity to bind to the VEGF over time by ELISA^9^, and did not study any of the physicochemical attributes of ziv-AFL. Although more research has been done on AFL (Eylea), few analytical studies have been carried out and most of these have focused on various physicochemical and functional properties of AFL in pharmaceutical compounding in prefilled syringes^10^. To the best of our knowledge, no forced degradation studies of ziv-AFL (Zaltrap) have been described to date. These studies are essential in that they are an integral part of biotherapeutics research and development and serve a variety of objectives ranging from early stage manufacturability evaluation to supporting comparability assessments both prior to and after approval for sale^11^. Forced degradation studies are also of great importance for quality control of these drugs after compounding in routine hospital use before administration, as they can test the impact of handling of the drug on its stability from the time it is released by the manufacturer up until its administration to patients^12^. In the most similar previously published paper, researchers studied AFL (Eylea) under physiological conditions as well as when incorporated into drug delivery systems^13^, and analysed the changes it undergoes when submitted to different temperatures and pH values. Although this paper presented valuable results, it did not focus on degradation studies.

It is well known that the degradation of recombinant therapeutic proteins has a negative impact on product quality, safety and efficacy and must therefore be detected when it occurs. Forced degradation studies can provide an in-depth understanding of the physicochemical and functional properties of the proteins, including the identification of the major degradation pathways that could not be observed in stability studies performed in real time^11^. In general, forced degradation studies are conducted by submitting a particular biopharmaceutical product to a range of experimental stress conditions. This conditions should avoid applying excessive or too little stress^14^. However, there are no practical protocols available for the preparation of stress tests on bio-pharmaceuticals nor there are well-established acceptance criteria to interpret the results obtained^15^. As a result, the particular stress tests and the conditions applicable in each one are normally decided by researchers themselves on the basis of their previous experience. These researchers are normally from academic institutions and biopharmaceutical companies.

In this paper, using an appropriate set of physicochemical and functional techniques, we perform a comprehensive analytical characterization of ziv-AFL (Zaltrap) and a forced degradation study in order to expand our knowledge of this complex Fc-fusion protein. We present, for the first time, some interesting results about this widely used Fc-protein that could also be very useful for evaluating the risks associated with its handling.

## Results

Ziv-AFL medicine (Zaltrap) was submitted to six forced degradation conditions: (i) exposure to high temperature (60 °C and 70 °C) for 1, 2 and 3 hours, (ii) exposure to light irradiation (250 W/m^2^) in an aging chamber (Solarbox 3000e RH, Cofomegra), (iii) 1 and 2 freeze-thaw cycles, (iv) pH = 5.2 and pH = 7.2, (v) exposure to a hypertonic medium, NaCl 1.5 M, and (vi) exposure to denaturing conditions by diluting ziv-AFL in GndHCl 8 M. A thermal stability study was performed by Circular Dichroism from 20 to 90 °C. There are no official documents explaining how to conduct forced degradation studies, except for the test involving exposure to light irradiation^16^. The stress study was therefore designed by considering a range of stress factors to which the medicine might be exposed in real-life conditions. In addition, GndHCl stress was used to study ziv-AFL in denatured conditions.

The aliquots were analysed using an array of methods to measure possible changes in the structure of ziv-AFL. A physicochemical and functional characterization of ziv-AFL was performed. A soluble particulates profile (dynamic Light Scattering –DLS- and analytical Size Exclusion Chromatography -SEC-), a conformational study of secondary and tertiary structures (circular dichroism –CD- and Intrinsic Tryptophan Fluorescence -IT-FS- spectroscopies) and a functional assay (ELISA) were also conducted.

### Visual inspections

The samples remained clear after the stress tests had been performed. No precipitates or particulate matter could be detected with the naked eye, except for the sample subjected to temperature stress at 70 °C. The exposure of ziv-AFL medicine to a temperature of 70 °C for 1 h caused the formation of a viscous opalescent material. This sample was analysed by ThT and Congo Red experiments in order to confirm whether it was an amyloid-like structure (Supplementary Data, Fig. 1).

### Circular dichroism (CD)

**Figure 1:**
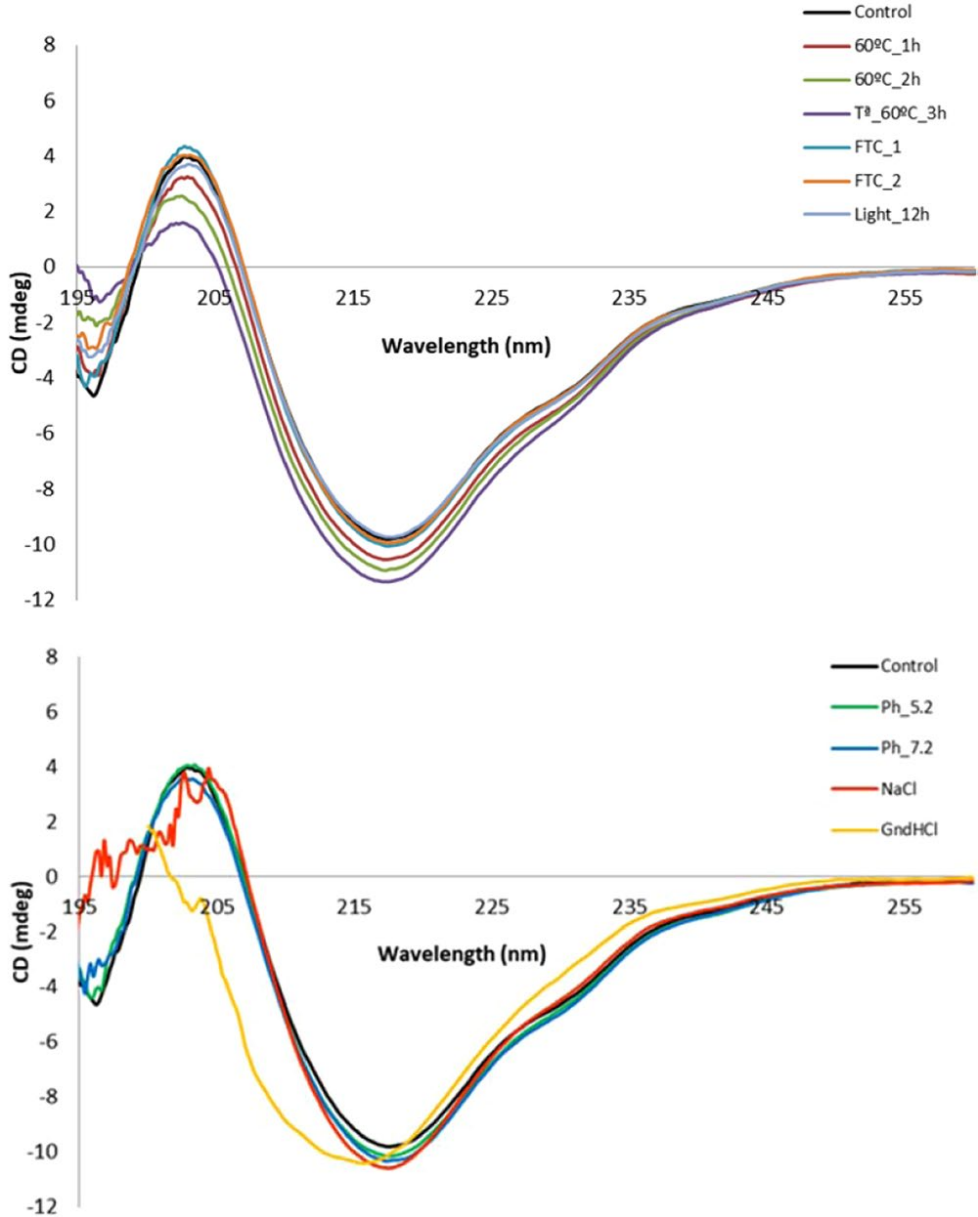
Far-UV CD spectra of all the stressed samples compared with ziv-AFL control samples (black line).

Alterations in the secondary structure are one of the first indicators of protein inactivation when exposed to different conditions^13^. Several ziv-AFL (Zaltrap, 25 mg/mL) fresh samples were diluted to 0.25 mg/mL and characterized by Far UV CD. At this concentration Ziv-AFL has the characteristic Far UV spectrum shown in Fig. 1 which was characterized by a wavelength of 207 ± 0.1 nm with ellipticity = 0; wavelength of the minimum at 217.5 ± 0.3 nm and a shoulder at around 229 ± 0.2 nm. These three checkpoints in the spectra were tracked in the stressed samples in order to detect changes in the secondary structures of ziv-AFL and the results obtained are presented in Table 1. The wavelength corresponding to ellipticity = 0 was considerably modified in the GndHCl stressed sample, a change that was expected as it was used as a positive control for protein denaturalization. This wavelength was also significantly altered when the sample was subjected to temperature stress, and slightly in the pH 7.2 and NaCl 1.5 M stresses. The wavelength of the minimum (217.5 nm) remained unchanged except in the case of GndHCl stress. The shoulder at 229 nm was modified in the GndHCl stress test. Ziv-AFL was only partially denatured by GndHCl as indicated by the CD spectra (Fig. 1). This was probably due to the dilution made prior to recording the CD spectra, when the sample was tested at the analysis concentration (ziv-AFL 0.25 mg/mL) in a similar GndHCl medium as the medicine was (GndHCl 8 M), complete denaturalization of the protein was indicated by the flat, featureless CD spectra. Ellipticity was additionally monitored at 215 nm in order to detect aggregation processes: 60 °C stress and GndHCl 8 M stress caused the greatest signal drop followed by NaCl 1.5 M, then by pH 7.2 and pH 5.2. The tests that caused the least stress were the F/T cycles and exposure to light. Nevertheless, aggregation was studied in greater depth with DLS and SEC.

**Table 1:** Secondary structures of ziv-AFL samples tracked by changes in the Far UV CD spectra.

Stress	Wavelength (nm) (Ellipticity = 0)	Minimum (nm)	Broad shoulder (nm)	Ellipticity at (215 nm)
Control	207.0 ± 0.1	217.5 ± 0.3	229 ± 0.2	−9.2
60 °C 1 h	***206.5***	217.4	228.8	−10
60 °C 2 h	***205.9***	217.4	229.0	−10.4
60 °C 3 h	***205.2***	217.4	229.0	−10.8
FTC 1	207.0	217.4	228.6	−9.4
FTC 2	207.1	217.6	228.8	−9.3
pH 5.2	206.9	217.6	229.0	−9.6
pH 7.2	206.7	217.4	228.8	−9.7
Light 12 h	206.9	217.8	228.6	−9.1
GndHCl 8 M	***201.7***	***215.6***	***230***	−10.4
NaCl 1.5 M	207.2	217.4	229.2	−10

Italic and bold: datum with significant difference respect to control.

Thermal stability was checked by applying a ramp from 20 °C to 90 °C. The CD spectra remained visually unaltered up to 60 °C (Supplementary Data, Fig. 2). Temperatures over 60 °C caused notable spectral changes. A percentage estimation of secondary structures showed a decrease in β-sheet structures and an increase in unordered structures.

The secondary structure content estimated from the CD spectra after mathematical deconvolution reflected a majority of β strands and random coil conformation, in line with the IgG1 based-structure of ziv-AFL^17^ (Supplementary Data, Table 1). Changes indicated above in the CD spectra were not clearly transferred to the secondary structure estimated by this approach.

### Dynamic light scattering (DLS)

DLS results are shown in Table 2. The Ziv-AFL (Zaltrap, 25 mg/mL) medicine sample showed a single population of particles with a hydrodynamic radius (R_h_) of 10.1 nm and a polydispersity (P_d_) of 9.9%. Temperature was the only stress that increased the R_h_. Moreover, after 2 h and 3 h of exposure the monodisperse population was split into two with P_d_ also increasing significantly. The R_h_ and P_d_ remained unaltered in all other stress conditions tested.

**Table 2:** Average hydrodynamic radius (R_h_) and polydispersity (P_d_) of ziv-AFL samples.

Concentration (mg/ml)	Stress	R_h_	Pd (%)
25 mg/mL	Control	10.1 ± 0.4	9.9 ± 6.4
	60 °C 1 h	***15.8***	***43.0***
	60 °C 2 h	***13.6***	10.8
		40.8	15.9
	60 °C 3 h	***17.8***	12.4
		49.1	19.8
	FTC 1	10.0	10.0
	FTC 2	10.1	10.7
	pH_5.2	—	—
	pH_7.2	10.3	11.6
	Light stress 12 h	9.9	12.4
0.6 mg/mL	Control	5.4 ± 0.3	16 ± 4.9
	GndHCl	***8.3***	11.9
	NaCl	5.2	12.1

Italic and bold: datum with significant difference respect to control.

In order to subject Ziv-AFL medicine (Zaltrap, 25 mg/mL) to the stress of GndHCl and NaCl media, samples were diluted in distilled water to 0.6 mg/mL. Fresh samples of ziv-AFL at this concentration were analysed as control. These samples showed a single population with an average R_h_ of 5.4 ± 0.3 nm and a P_d_ of 16% (Table 2). Dilution therefore dramatically reduced the R_h_, while increasing the P_d_. In the samples stressed with GndHCl the R_h_ increased significantly. When the samples were subjected to NaCl stress, the R_h_ and P_d_ remained unaltered.

### Intrinsic tryptophan fluorescence spectroscopy (IT-FS)

Significant changes can take place in the tertiary structure of the protein without this causing much change in its secondary structure, although the long-term stability of the protein may be affected. These changes in the tertiary structure can expose some hydrophobic pockets to the solvent, which makes the protein itself more prone to aggregation. Ziv-AFL (Zaltrap, 25 mg/mL) was first characterized by IT-FS, which was used to calculate the spectrum centroid (or Centre of spectral mass -C.M-), which was 346.4 nm for fresh (control) samples. Table 3 shows the Centre of spectral mass (C.M) of the fluorescence spectrum of fresh (control) and stressed samples. GndHCl, temperature (60 °C) and light stresses caused the most significant increases in the C.M value, followed by pH, which induced slight increases. NaCl stress caused a fall in the C.M value. By contrast, G.C was unaltered when the samples were subjected to F/T stress.

**Table 3:** Ziv-AFL tertiary structure assessed by means of the Centre of spectral mass (C.M) of the fluorescence spectrum.

Stress	Centre of spectral mass (C.M) (fluorescence spectrum)
Control (25 mg/mL)	346.4
60 °C 1 h	348.0
60 °C 2 h	348.6
60 °C 3 h	349.0
FTC 1	346.4
FTC 2	346.4
pH 5.2	346.6
pH 7.2	346.5
Light stress	347.2
GndHCl	359.4
NaCl	345.9

### Size-exclusion high performance liquid chromatography with diode array detection (SE-HPLC/DAD)

Aggregation is most common form of protein degradation. With this in mind, fresh and stressed samples of ziv-AFL were analysed by SE-HPLC/DAD in order to obtain their SEC aggregates profile. As in the above studies, the ziv-AFL medicine was analysed immediately after opening the vial (fresh sample 25 mg/mL) and the resulting profile was used as a control for comparing with the chromatograms for stressed ziv-AFL samples in order to detect any changes.

**Figure 2:**
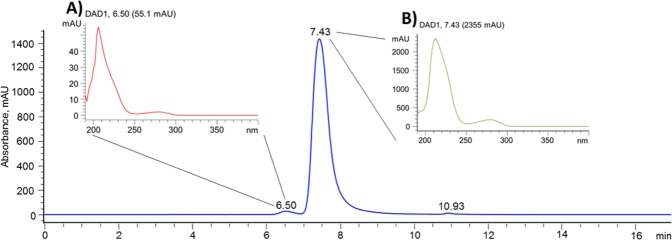
Representative SE/HPLC-DAD chromatogram for ziv-AFL 25 mg/mL (Zaltrap). UV spectra for the dimer population, peak at 6.50 (**A**) and for the monomer population, peak at 7.43 min (**B**).

The SE chromatogram for the control sample of Ziv-AFL (fresh medicine 25 mg/mL) is shown in Fig. 2. This control SEC aggregation profile displays two clear peaks. The corresponding molecular weight (MW) for each peak was calculated by using the estimated calibration model for the size-exclusion column (Supplementary Data, Figs. 3 and 4). The main peak, at 7.42 ± 0.02 min (98.1% ± 0.02% of the total area) was assigned to ziv-AFL monomers with an estimated MW of 164 kDa. A small peak eluting at 6.5 ± 0.01 min (2% ± 0.02% of the total area), just before the monomer, was assigned to natural and soluble aggregates, which due to their estimated MW of 358 kDa seem likely to be dimers.

In size-exclusion chromatography (SEC) the separation of molecules depends on their hydrodynamic volume and not on their molecular weight. The retention time is therefore affected by protein chain folding. In the case of ziv-AFL the retention time obtained for the monomers was shorter than expected for a molecule of its MW (115 kDa approx^2^) and, consequently, the estimated MW was higher (164 kDa). This result shows that the hydrodynamic volume of ziv-AFL is greater than the volume of the proteins used for the calibration of the column, which means that the “apparent mass” of ziv-AFL is higher than that indicated by the manufacturer^2^. A similar MW (150 kDa) was obtained previously for AFL (EyLea, 40 mg/mL, Bayer Pharma AG, Germany) by SDS-Page^13^. Ongoing research by high resolution mass spectrometry using different approaches has confirmed the indicated MW of 115 kDa approx^2^. Also, these current investigations have brought out the great complexity of the glycan profile that could be the responsible of the volume greater than that expected by the only considering its MW in SEC.

In addition, the peaks of both the monomers and the dimers exhibit similar UV spectra and a high degree of spectral peak purity (>99%) (in Fig. 2A,B. respectively).

**Figure 3:**
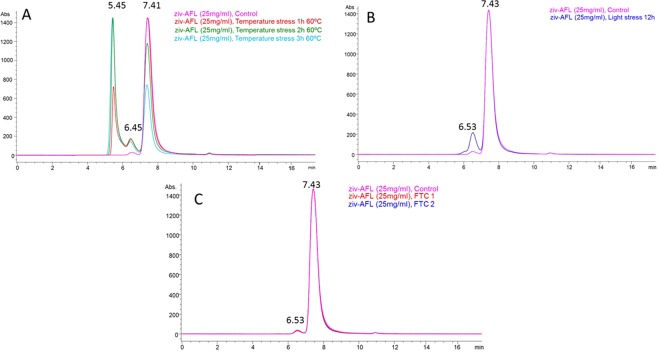
SE/HPLC-DAD chromatograms for the samples of ziv-AFL (Zaltrap 25 mg/mL) subjected to physical stress. **(A)** Ziv-AFL Temperature stress 60 °C: (1) ziv-AFL control, (2) 1 hour of exposure, (3) 2 hours of exposure and (4) 3 hours of exposure. **(B**) Ziv-AFL light stress: (1) Ziv-AFL control and (2) 12 hours of exposure. **(C)** Ziv-AFL freeze-thaw stress: (1) Ziv-AFL control, (2) 1 freeze-thaw cycle and (3) 2 freeze-thaw cycles. The retention times represented in the SEC chromatograms are the mean of three chromatograms.

**Figure 4:**
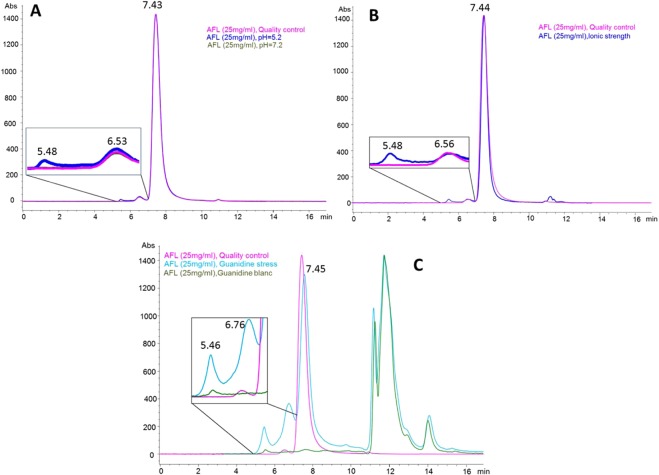
SE/HPLC-DAD chromatograms of samples of ziv-AFL (Zaltrap 25 mg/mL) subjected to chemical stress. **(A**) Ziv-AFL pH stress: (1) Ziv-AFL control, (2) pH = 5.2 and (3) pH = 7.2. **(B**) ionic strength stress: (1) Ziv-AFL control and (2) NaCl 1.5 M. **(C**) Ziv-AFL GndHCl stress (1) Ziv-AFL control, (2) GndHCl 8 M stressed samples and (3) GndHCl 8 M, blank sample. The retention times represented in the SEC chromatograms are the mean of three chromatograms.

As regards the stress study, Figs. 3 and 4. show the SEC chromatograms under physical and chemical stress conditions respectively. The retention times and percentage of the peak detected in the chromatograms are indicated in Table 4. Ziv-AFL samples submitted to 60 °C degraded gradually over time into two main entities (Fig. 3A, Table 4). The first was a dimer (6.5 min) and the second was an aggregate of about 10 ziv-AFL molecules with an estimated MW of 1666 kDa (5.45 min). The degradation promoted by light exposure (12 hours) led to the transformation of 10.5% of the monomers into dimers in 12 hours (Fig. 3B, Table 4). No changes were detected when the ziv-AFL samples were submitted to freeze-thaw cycles (Fig. 3C) and the percentage of monomers and dimers remained constant (Table 4).

**Table 4:** Overall results for experimental retention time and abundance in the SEC analysis.

Stress	Monomer		Dimer		Non-natural HMW aggregates	
	Retention time (min)	Abundance (%)	Retention time (min)	Abundance (%)	Retention time (min)	Abundance (%)
Control	7.43	98.1	6.50	1.9	—	—
60 °C 1 h	7.42	69.9	6.48	8.0	5.48	22.1
60 °C 2 h	7.40	52.1	6.46	7.8	5.45	40.1
60 °C 3 h	7.40	37.1	6.42	7.7	5.43	55.2
Light 12 h	7.43	87.6	6.53	12.4	—	—
FTC 1	7.42	98.1	6.54	1.9	—	—
FTC 2	7.43	97.9	6.52	2.1	—	—
pH 5.2	7.43	97.2	6.53	2.0	5.48	0.8
pH 7.2	7.43	98.2	6.53	1.8	—	—
NaCl 1.5 M	7.44	97.5	6.56	1.4	5.48	1.1
GndHCl 8 M	7.45	35.6	6.76	19.0	5.46	7.2

Moreover, increasing the pH by one unit (up to 7.2) compared to the original value for the medicine (6.2) did not promote ziv-AFL degradation as no changes were detected in the SEC profiles (Fig. 4A). By contrast, a more acidic pH (5.2) did lead to aggregation (Fig. 4A), with a slight increase in the dimers at 6.50 min and the detection of the HMW aggregate entity at 5.48 min, albeit representing a small percentage (0.8%). Similar degradation of ziv-AFL was detected in a high ionic strength medium, with a slight decrease  in the dimers (at 5.6 min and 1.4%) and the detection of the higher order aggregation population (at 5.48 min and 1.1%) as shown in Fig. 4B and Table 4.

As expected, the presence of GndHCl in the medium (Fig. 4C) promotes high level ziv-AFL degradation; the main chromatographic peak at 7.45 min (monomers) decreased significantly (around 62.5%) in parallel with the increase in the dimers at 6.76 min (17.1%) and in the entity already detected when ziv-AFL was subjected to other stress conditions, namely a higher order of aggregation at 5.46 min (5.56%). This SEC chromatographic profile shows 2 zones: the zone where ziv-AFL and its aggregates elute (from 0 to 10 min) and the zone where the reactive elutes (from 10 to 17 min).

A chromatographic peak was detected in all the SEC profiles at a retention time of 10.92 ± 0.02 min, including those from the control samples. We therefore assumed that this was due to some of the excipients used in the formulation of the medicine.

### ELISA

**Figure 5:**
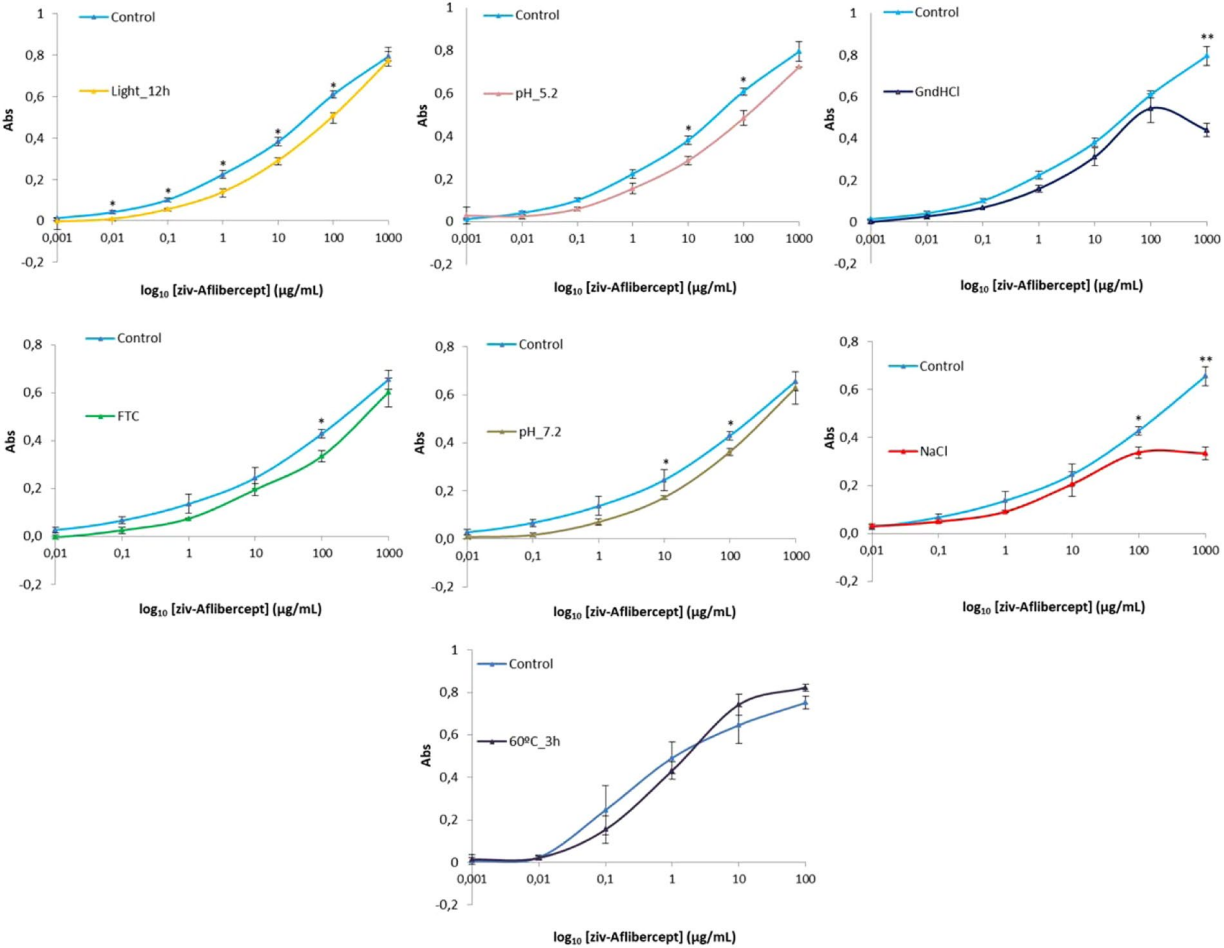
ELISA binding assay (1 ng/mL–1000 µg/mL) for all the stressed samples represented with respect to a fresh (control) sample of ziv-AFL (Zaltrap 25 mg/mL).

ELISA-based binding assays are essential for studying therapeutic proteins. They offer a direct way of evaluating biological activity (antibody-antigen binding specificity) and thus help us understand the impact of the physicochemical changes in these proteins on the effectiveness of the biotechnological medicines. Figure 5 shows the results of the ziv-AFL VEGF ELISA. The absorbance is represented as a function of the concentrations of ziv-AFL after dilutions of the control sample and the particular stressed sample, so enabling the results to be visually compared using the graphs (Fig. 5). A Student’s t analysis was carried out for the means comparison in order to determine whether the results of the stressed sample are significant or whether there are any changes compared to the control samples (Fig. 5) (those with asterisks are significant). Unexpectedly, ziv-AFL retains important functionality as a medicine after undergoing most of the stress tests. A clear decrease in functionality as manifested in a loss in VEGF binding capacity was only observed in the samples subjected to light-exposure stress. In the GndHCl and NaCl medium test, a clear deviation of the function was only observed in the most concentrated test sample. This was also the least diluted sample (factor of 25), which could explain the negative effect that the stress medium itself had on the ELISA process. The results were not clear when samples were stressed with a pH of one unit more or less than the pH of the ziv-AFL medicine solution (6.2); results for the test samples diluted at 10 and 100 µg/ml were significantly different from the control with an estimated remaining biological activity of around 25% (pH 5.2) and 36% (pH 7.2), while the other concentrations (dilutions) tested in the ELISA test did not produce significant results, i.e. indicating no loss of biological activity compared to the control samples. In this case, the high dilution performed on the stressed samples, i.e. dilution factors of 250 and 2500 for 100 and 10 µg/ml respectively, cannot explain the results of the ELISA test. It is therefore possible that changes in the pH cause a loss of functionality by decreasing the ziv-AFL binding capacity.

## Discussion

Ziv-AFL is an anti-VEGF antibody-based drug synthetized by the Chinese Hamster Ovary (CHO) cell. It is a glycosylated fusion protein which has a protein structure with a molecular weight of 97 kDa. According to the manufacturer, the glycosylation part increases the weight of the molecule (115 kDa) by 15%^3^.

Ziv-AFL (Zaltrap, 25 mg/mL) medicine is composed of monomers and natural dimers which make up 2% of the total molecules. This was demonstrated by the SEC profiles (Fig. 2). The spectral peak purity for each entity was found to be over 99%, with a high level of uniformity of a single compound in the chromatographic peaks, with no impurities. The CD spectrum is characterized by a minimum at 217.5 nm, a wavelength for ellipticity = 0 of around 207 nm, and a shoulder at around 229 nm. The CD spectral features indicate that the protein mainly consists of a β-sheet structure (Supplementary Data, Table 1), in accordance with the IgG1 structure. The tertiary structure is characterized by intrinsic tryptophan fluorescence spectroscopy with a centre of spectral mass (C.M) of 346.4 nm (Table 3). Undiluted medicine (25 mg/mL) shows an R_h_ of 10.1 nm and a polydispersity of 9.9%, while diluted ziv-AFL medicine (0.6 mg/mL) presents an R_h_ of 5.4 nm and a polydispersity of 16% (Table 2).

**Figure 6:**
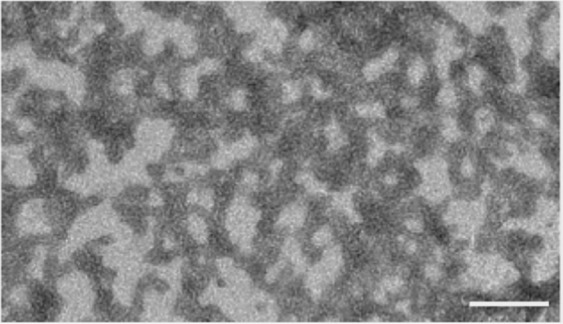
TEM image of a freshly prepared 1.0 mg·mL^−1^ ziv-AFL sample subjected to 70 °C for 1 h. The white segment corresponds to 200 nm.

Exposure to high temperatures induced aggregation in the ziv-AFL medicine samples. This fact was corroborated by the results obtained using the different techniques. SE-HPLC demonstrated that aggregation increases proportionally in line with the time of exposure to heat (Table 4). The most interesting finding was the peak detected at 5.45 min retention time (Fig. 3A) These HMW aggregates (around 1666 KDa) is related to ß-amyloid structures, in that a progressive increase of the negative band in the CD spectroscopy was observed at 215 nm. This phenomena has been widely studied and correlated with this specific form of aggregation^18^. where protein secondary structure elements reorganize to finally give rise to extensive β-structure, common in amyloid fibrils^19^. Moreover, the amyloid nature of the aggregates was confirmed by a very significant increase in the thioflavine (ThT) fluorescence signal and a red-shift of the Congo red absorbance induced by the aggregates (Supplementary Data, Fig. 1). These two assays are standard for amyloid fibril characterization^20,21^ *in vitro*. The formation of these non-native aggregates in many proteins has also been linked to exposure to high temperatures^22^. Finally, the fibrillary nature of this HMW aggregate was observed by Transmission Electron Microscopy (TEM)^20^ (Fig. 6); TEM image clearly shows the presence of curly amyloid protofibrils, thus confirming both the ThT and Congo Red analyses results. The secondary structure of the protein was also modified. The negative band mentioned above decreased progressively over time during exposure to heat and the wavelength with ellipticity = 0 shifted to the far UV. However, all these changes did not result in alterations in the percentage of secondary structures (Supplementary Data, Table 1) perhaps due to the lack of sensitivity of the mathematical algorithm for detecting CD spectra modifications. The tertiary structure was also altered, as manifested in the red shift observed in IT-FS. Contrary to what might be expected, all these physicochemical changes do not appear to affect the ziv-AFL-VEGF binding, as indicated by the ELISA results (Fig. 5) and therefore, the biological activity of the medicine in solution is maintained despite the presence of these HMW aggregates.

The 12 h light stress also caused ziv-AFL aggregation. It is well known that exposure to light induces aggregation in monoclonal antibodies^23,24^, and the same occurs in the ziv-AFL medicine samples, in which the dimer population clearly increased progressively over time during light exposure (from 1.9% to 12.2% (quantified by SE-HPLC). However, this increase in the dimer population was not detected by DLS. This may be due to the low sensitivity of this technique compared to analytical SEC. Light is also recognized as an oxidizing agent^23^. It is known that light oxidizes tryptophan residues on biopharmaceutics with the subsequent loss of biological activity^25^. Aggregation of IgG accompanied by loss of biological activity has also been previously reported^25^. In our research, this stress did not affect the secondary structure content, although changes in the tertiary structure were detected. A significant red shift suggests a modification in the tryptophan environment. This indicated that the conformation of the protein changed as tryptophan residues become more exposed to the solvent. As regards functional properties, light degradation causes a decrease in the capacity to bind to VEGF. Therefore, exposure to light induces irreversible modifications in the protein conformation, which compromise its affinity for VEGF. In this case, the observed increase in the dimer population could be involved in this decrease in biological activity. It is also interesting to note that dimers were the highest orders of aggregation produced by light exposure.

No changes were noted in ziv-AFL after the freeze/thaw cycles. The monomer population remained constant throughout this stress test and the secondary and the tertiary structures also remained stable. For its part, the biological functionality of ziv-AFL measured in terms of its binding capacity to VEGF was not affected, although a slight, insignificant decrease was observed in the ELISA results (Fig. 5). Protein aggregation is the primary form of degradation in therapeutic proteins when subjected to this form of stress^26^. Freeze/thaw stress disturbs local structure on the surface of the residues leading to aggregation processes preceded by partial denaturation during freezing^27^. The two freeze/thaw cycles performed in this study were not sufficient to induce aggregation in ziv-AFL.

With regard to the NaCl stress, non-natural aggregation was observed by SE-HPLC at 5.48 min and was quantified as 1.1% of the total signal chromatogram. This small population was not however detected by DLS in which R_h_ and P_d_ remained unchanged, again due to the low sensitivity of this technique. In addition, the ellipticity of the negative band at 215 nm increased slightly, which means that these aggregates are the amyloid-like previously proposed. It has been reported that high ionic strength accelerates the protein aggregation process^28^. In this case the process begins in less than 24 hours. The secondary structures of the protein remained unaltered, although a conformational change was detected by IT-FS. A blue shift in the fluorescence emission spectra took place indicating that the tryptophan environment became more hydrophobic. Inorganic salts such as NaCl are known to favour compact protein conformations^29^ and can also affect their biological activity. In the ELISA tests, there was no binding at the highest protein test concentration assayed (1000 µg/mL). This concentration corresponds to an ionic strength of 1.44 M that could affect the ELISA method. With the serial dilutions and thus the elimination of Na^+^ and Cl^−^ ions from the medium, the affinity to VEGF was significantly recovered. This could indicate that hypertonic NaCl solution induces reversible conformational changes or that this high concentration of 1.44 M could be affecting the VEGF in the ELISA plates, not allowing ziv-AFL and VEGF to bind. Once again, the appearance –although at small percentage- of higher states of aggregation (around 1666 KDa) of the protein in the samples does not seem to be associated with a decrease in the capacity of ziv-AFL to bind to VEGF.

GndHCl is a strong denaturing chaotropic, widely used in conformational studies of macromolecules. As expected, this stress induced the most dramatic changes in the structural integrity of the protein. In fact, it was initially selected as a positive control for ziv-AFL degradation. As regards aggregation, an important increase was observed using SE-HPLC in the percentage of the dimer population (from 1.9% to 19%) together with the peak at 5.46 min (7.2%). This was corroborated by DLS in which R_h_ increased significantly from 5.4 nm (control sample) to 8.3 nm. The ellipticity of the negative band at 215 nm also increased, which suggests that these HMW aggregates are β amyloidal. As regards the conformation of the protein: secondary structures of the protein were significantly altered, with notable changes in the three parameters studied. An estimation of the percentage of secondary structures revealed a slight switch from characteristic β sheets structures to α helix. The tertiary structure of the protein was also modified. The C.M increased significantly indicating widespread modification of the tertiary structure. All these results were compared with those obtained by ELISA. Similarly, as occurred when the ziv-AFL medicine samples were subjected to NaCl stress, at the highest test concentrations analysed (1000 µg/mL ziv-AFL) in which the concentration of GndHCl in the medium was also the highest tested (1.28 M), a significant decrease in the capacity of ziv-AFL to bind to VEGF was observed. However, with dilutions and the consequent elimination of the stress agent from the medium, ziv-AFL bound to VEGF with a high affinity at all the test concentrations analysed. Unexpectedly, although all the other techniques reflect severe modifications in the structural integrity of ziv-AFL, functionality –measured in terms of its capacity to bind to VEGF- was maintained.

Therefore, these results suggest that the soluble β-amyloid aggregates, which are detected in all the stressed ziv-AFL medicine samples, except those exposed to light and those subjected to freeze/thaw cycles, could slightly increase the signal of the immunoassay by increasing the IgG-conjugate binding ratio. Despite to be initially unexpected, these results could be attributed to the AFL high binding capacity to its antigen, that even unfolding and/or degraded, AFL kept the binding capacity to VEGFR, It was demonstrated that the AFL’s equilibrium disassociation constant (Kd, inversely related to binding affinity) for VEGF-A165 was 0.49 pM, compared with 9.33 pM and 88.8 pM for native VEGFR1 and VEGFR2, respectively, then AFL binds with a greater affinity than the body’s native receptors^30^]. Then, it could occur that once this aggregate is attached to the VEGFR, more than one IgG-conjugated can bind to it, increasing the IgG-conjugate binding ratio for each ziv-AFL-VEGFR immunocomplex formed.

The ziv-AFL (Zaltrap, 25 mg/mL) solution has a pH of 6.2. Results suggest that increasing or reducing the pH by one unit caused slight but significant changes. pH 5.2 induced the formation of HMW aggregates (5.46 min) amounting to 0.8% of the total. The protein secondary structure content was maintained, although it was again observed that the negative band at 215 nm increased slightly, a change that could be due to the newly-formed HMW aggregates. There was a significant increase in the C.M value, suggesting a partial loss of ordered conformation. The isoelectric point of AFL is 8.2^31^, so pH 5.2 makes the molecules of ziv-AFL highly protonated, giving them a positive net charge. The increase in the positive charge within a monomer can increase the repulsion between amino acids with the same positive charge^28^, leading to conformational changes or partial denaturation, forcing hydrophobic pockets (hot spots) to be exposed and aggregation to begin. The ELISA assay reveals a general decrease in ziv-AFL affinity to VEGF. For its part, pH 7.2 did not cause aggregation detectable by either of the techniques used (SE-HPLC and DLS). This result is surprising given that increasing the medium pH to 7.2 brings it closer to the isoelectric point of the protein, so decreasing its solubility. The secondary structure of the protein remained the same, a result obtained by comparing the CD spectrum for this sample with that for the control. The C.M was also modified as denoted by a slight conformational change in the protein, which in turn could explain the observed decrease in the capacity of ziv-AFL to bind to VEGF at this pH value.

## Conclusions

There are many stresses to which therapeutic proteins might be exposed during handling, both during the manufacturing process and later in hospital. The results presented here indicate that Zaltrap should be stored away from the light. Exposure to light causes significant inactivation of this drug, which could render the treatment ineffective. Light also causes dimerization, that in agreement with many published work^23^ it is promoted by chemical degradation (mainly oxidations) that could compromise the safety and efficacy of the medicine. Storage at 4 °C is also necessary. The higher the temperature the more prone the protein is to form aggregates, including not only dimers but also higher order aggregates characterised by β-amyloid-like structure. The biological activity of the protein - measured by means of the ziv-AFL binding capacity to VEGFR- in solution was maintained at 60 °C, despite the appearance of HMW soluble aggregates, although they are likely to impair the safety and the efficacy of the medicine. Caution must also be taken when diluting the medicine in NaCl since a highly hypertonic solution also causes HMW aggregates to appear –although in small proportion-. Increasing the ionic strength in the solution could therefore initiate aggregation during storage. It is also important to control and maintain the pH in the medium, as pH shifts can cause partial inactivation of the molecules, possibly due to conformational changes. We also found that a pH of about 5.2 initiates aggregation processes leading to HMW. Finally, ziv-AFL seems to be relatively resistant to F/T cycles (2 in this case). However, exposure to several repeated F/T cycles is not recommended as it could eventually promote aggregation and therefore inactivation of the medicine. As expected, this research clearly demonstrates the fragile nature of biotechnological drugs and in particular of the fusion protein ziv-AFL, with the detection of a soluble ziv-AFL HMW aggregate related to an amyloid-like structure.

## Materials and Methods

### Materials

Zaltrap^,^ 25 mg/ml vials (Sanofi-aventis groupe) were supplied by the University Hospital San Cecilio (Granada, Spain) within the framework of the current project FIS17-00547, Instituto Carlos III, Ministerio de Economía y Competitividad, Spain.

### Forced degradation

Forced degradation studies were performed on the medicine Zaltrap by subjecting the samples to the particular stress condition and then storing the stressed samples for 24 h (at 4 °C and protected from the light) before analysis. Six forced degradation conditions were tested: (i) exposure to a temperature of 60 °C for 1 h, 2 h and 3 h, and subjecting one sample to a temperature ramp from 20 °C to 90 °C (for CD analysis) (ii) exposure to light irradiation (250 W/m^2^) for 12 h in an aging chamber (Solarbox 3000e RH, Cofomegra, Milan, Italy)^16^, (iii) two freeze-thaw cycles, (iv) exposure to an acidic medium by adjusting pH to 5.2 by adding 0.1 M of HCl and to a basic medium by adjusting pH to 7.2 by adding NH_3_ 1 M (Sigma-Aldrich, USA), (v) exposure to a hypertonic medium by diluting ziv-AFL in 1.5 M NaCl at a final concentration of 0.6 mg/ml, and (vi) exposure to denaturing conditions by diluting ziv-AFL in GndHCl 8 M (pH 8.5, Sigma-Aldrich, USA) at a final concentration of 0.6 mg/ml; this GndHCl medium was also used as a positive control for ziv-AFL degradation.

When possible, all the samples, control samples and those subjected to stress, were analysed without dilution, i.e. in ziv-AFL medicine format (25 mg/mL). This was to make sure that the aggregation equilibrium was not altered by dilution. In the CD study, the samples had to be diluted at a drug concentration of 0.25 mg/mL (so as to avoid detector saturation). The only exception was the GndHCl stressed sample, which was analysed at 5 mg/mL.

### Far UV circular dichroism (CD) spectroscopy

The experimental conditions were similar to those used in ref. ^32^. Spectra were recorded using a JASCO J-815 spectropolarimeter (JASCO, Tokyo, Japan) equipped with a Peltier system for temperature control. Temperature was set at 20 °C for all measurements. Solution samples were studied at the target concentration of 0.25 mg/ml. Spectra were acquired every 0.2 nm with a scan speed of 20 nm/min from 260 to 190 nm and a total of 5 accumulations were averaged, with a bandwidth of 1 nm. A 1 mm path length quartz cuvette was used throughout. The blank was first measured and subtracted from the samples. Means-Movement Smoothing was applied to all the spectra with Spectra Analysis software. Spectra were exported as ASCII files, and then secondary structures were estimated using CDSSTR^33^ and CONTINL^34^ algorithms and SP175 protein DataSet^35^ available at the Dichroweb website^36^.

### Intrinsic tryptophan fluorescence spectroscopy

The experimental conditions were similar to those used in ref. ^32^. Measurements were carried out on a Cary Eclipse spectrofluorometer (Agilent, Santa Clara, CA, USA) equipped with a Peltier system for temperature control. Excitation wavelength was set at 298 nm and emission was recorded from 310 to 400 nm. Solution samples were not diluted before measuring and temperature was set at 20 °C. Spectra were recorded at a scan speed of 600 nm/min and a total of 5 spectral accumulations were acquired. Excitation and emission slits were initially set at 5 nm. Excitation slits were modified depending on the samples in order to avoid saturation or low fluorescence signal. Data centre of spectral mass (C.M) was calculated for each spectrum using Eq. 1:

1 $${\rm{C}}{\rm{.M}}=\frac{{\sum }_{{\rm{i}}=1}^{{\rm{n}}}({{\rm{f}}}_{{\rm{i}}}{{\rm{\lambda }}}_{{\rm{i}}})}{{\sum }_{{\rm{i}}=1}^{{\rm{n}}}{{\rm{f}}}_{{\rm{i}}}}$$

where f_i_ is the fluorescent intensity and λ_i_ is the wavelength.

### Dynamic light scattering (DLS)

The experimental conditions were similar to those used in ref. ^32^. DLS measurements were carried out in a Protein Solutions DynaPro-99 System Dynamic Light Scattering Module equipped with a Temperature Control Micro Sampler (Wyatt, Santa Bárbara, California, USA). A 1.5 mm path length quartz cuvette, which was cleaned thoroughly before measuring, was used throughout. A minimum of 100 reads were recorded per measurement. Temperature was set at 20 °C and the acquisition time was 5 s. Laser power was lowered depending on the concentration of the samples. Solvent parameters were also modified according to each sample.

### Size exclusion chromatography (SE/HPLC-DAD)

The analysis was performed by liquid chromatography using an Agilent 1100 chromatograph equipped with a quaternary pump, degasser, autosampler, column oven and photodiode array detector (Agilent Technologies). Drug chromatographic evaluation was carried out in a SEC-5 column (300Bio, Agilent Technologies, USA) in which temperature was set at 25 °C. The flow rate was 0.35 mL/min and the mobile phase was composed of 150 mM of phosphate buffer pH 7.0, which was prepared with anhydrous disodium hydrogen phosphate (Panreac, Spain) and monohydrate monobasic sodium phosphate (Sigma-Aldrich, USA). An isocratic elution mode was applied for 17 min. 1 µl of sample was injected into the column. The UV-visible spectra were recorded between 190–400 nm with a data point every 0.5 nm. Chromatograms were registered at 214 nm with a reference band at 390 nm.

The column was calibrated in order to establish the relationship between the molecular weight and the retention time of the Fc-protein. The calibration kit (Agilent, USA) was composed of 5 proteins: thyroglobulin (670 kDa), γ-globulin (150 kDa), ovalbumin (45 kDa), myoglobin (17 kDa) and angiotensin II (1 kDa). The chromatogram and the experimental retention times for the calibration kit are shown in the Supplementary Data provided (Supplementary Data, Fig. 4) (molecular weight = −0.4493x^2^ + 2.1667x + 8.7161; R^2^ = 0.9957). The calibration model was used throughout this study.

### Functional-based method: enzyme-linked immunosorbent assay (ELISA)

This method was here optimized for ziv-AFL, starting from the outline of that optimized for infliximab^32^. An indirect and non-competitive ELISA based on the specific interaction of ziv-AFL with VEGF was carried out to test the biological activity of ziv-AFL after the various stress factors had been applied. 96-well Maxisorp immune plates were sensitized with VEGF by incubation overnight (18 h) at 4 °C adding 100 μL/well of 0.25 μg/mL VEGF diluted in 0.1 M carbonate buffer solution (pH 9.6). The plates were washed (VASHER RT-2600C microplate washer, Comecta, Abrera, Barcelona) four times with PBS-Tween 20 (pH 7.4 containing 0.3% (v/v) Tween 20). They were then treated with 100 μl of the blocking buffer (PBS pH 7.4 containing skimmed milk 2% (w/v)) per well for 2 h at 37 °C to eliminate nonspecific absorptions. The plate was again washed and filled with 100 μl of ziv-AFL appropriately diluted in 0.1 M carbonate buffer pH 9.6 at several concentrations ranging from 1 ng/mL to 1000 μg/mL. After incubating for 45 min at 37 °C, the plate was washed four times with PBS and incubated again with 100 μl/well of a solution of 10 μg/mL of peroxidase-labelled rabbit anti-human IgG prepared in 0.1 M carbonate buffer solution with a pH of 9.6 for 30 min at 37 °C. After washing four times with PBS, 100 μl of the substrate solution (OPD) was added to each well and incubated for 20 min in darkness at room temperature (25 °C). The reaction was stopped by adding 50 μl of 1 M sulfuric acid solution. Absorbance was recorded at 450 nm and 620 nm, and the analytical signal was the difference between the two absorbance values (TECAN SUNRISE™ microplate absorbance reader for 96-well plates connected to the computer running XFluor4 software, Tecan, Austria, GMB.

### Congo red binding assay

The experimental conditions were similar to those used in ref. ^20^. 70 °C sample was tested for amyloid-specific Congo red binding by the spectroscopic band shift assay. Aliquots of protein solution: control and stress (70 °C) (6 μl) were diluted in the reaction buffer (150 mM potassium phosphate, 150 mM NaCl, pH 7.4), which contained 20.8 μM Congo red (150 µl final reaction volume). The Congo red solution was freshly prepared and filtered through a 0.2 μm filter before use. The absorption spectra were acquired after 2–3 min equilibration using a Cary 50 BIO UV-VIS spectrophotometer (Agilent, Santa Clara, CA, USA).

### Thioflavin T binding assay

The experimental conditions were similar to those used in ref. ^20^. ThT binding assays were performed as described^20^ using a Cary Eclipse spectrofluorometer (Agilent, Santa Clara, CA, USA). ThT was excited at 440 nm with a 5 nm slit width and the fluorescence emission was recorded at 485 nm with a 5 nm slit width. A 250 μM stock solution of ThT was freshly prepared in 25 mM potassium phosphate (pH 6.0). Protein aliquots (80 μl) were diluted into water (110 µl) and the phosphate buffer containing 250 μM ThT (10 µl) to a final volume of 200 µl. Measurements were carried out at room temperature using a 3 mm pathlength cuvette.

### Transmission electron microscopy

The experimental conditions were similar to those used in ref. ^20^. For transmission electron microscopy (TEM), protein samples were diluted ten times with water. The samples (15 mL) were then placed on a formvarcarbon-coated copper grid and allowed to stand for 4 min. The grid was then washed twice with distilled water and stained with 1% uranyl acetate for 1 min. The dried samples were evaluated in a Zeiss 902 electron microscope (Zeiss, Oberkochen, Germany) operating at an accelerating voltage of 120 kV and observed at a magnification of 50000.

## Supplementary information

Supplementary data.

## Data Availability

The datasets generated and/or analysed during the current study are available from the corresponding author on reasonable request.
