# Supplementary material for: Comprehensive biophysical and functional study of ziv-aflibercept: characterization and forced degradation
Source: Sci Rep. 2020 Feb 14;10:2675. doi: 10.1038/s41598-020-59465-7 (PMC7021693; doi:10.1038/s41598-020-59465-7)
Supplement: Supplementary file 1 — Supplementary data. [file 41598_2020_59465_MOESM1_ESM.docx]

**SUPPLEMENTARY DATA**

**Comprehensive biophysical and functional study of ziv-aflibercept: characterization and forced degradation**

Jesús Hermosilla^a^, Raquel Pérez-Robles^a^, Antonio Salmerón-García^b^, Salvador Casares^c^, Jose Cabeza^b^, Jonathan Bones^d^, Natalia Navas^a^^[[1]](#footnote-1)^🖂

^a^ Department of Analytical Chemistry / Institute for Biomedical Research (ibs.GRANADA), University of Granada, E-18071 Granada, Spain

^b^ Department of Clinical Pharmacy, San Cecilio University Hospital, Institute for Biomedical Research, (ibs.GRANADA) 18016 Granada, Spain

^c^ Department of Physical Chemistry / Institute of Biotechnology, University of Granada, E-18071 Granada, Spain

^d^ Characterisation and Comparability Laboratory, NIBRT. Fosters Avenue, Mount Merrion. Blackrock, Co. Dublin, Ireland

*Supplementary Data*

*ThT and Congo red binding assays*


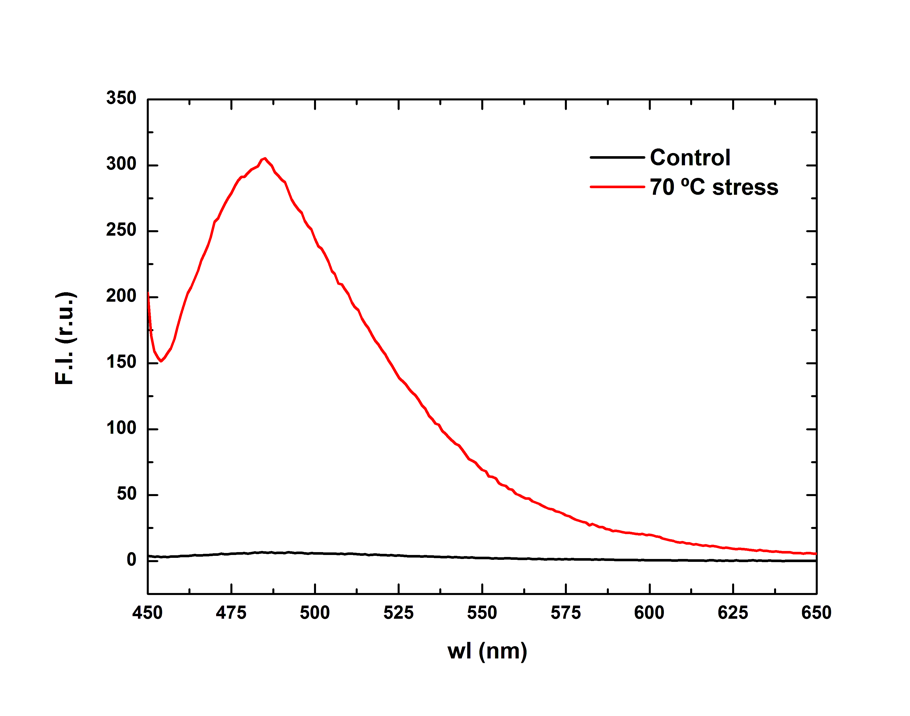

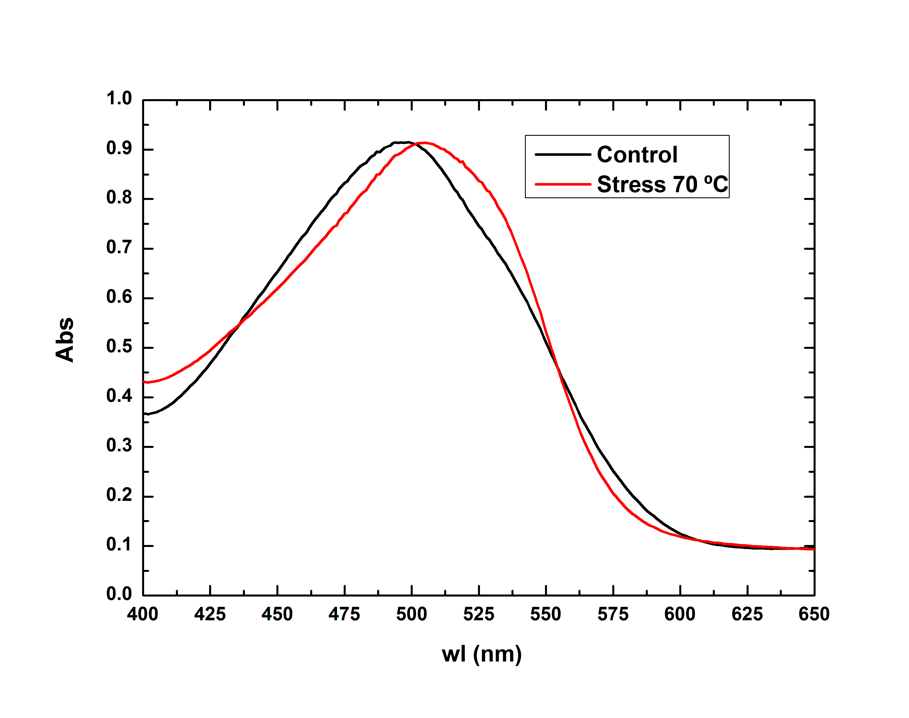


**Figure 1**: Standard characterization of the amyloid-like aggregates observed when a 25 mg/mL fresh ziv-AFL sample is heated up to 70ºC for 1 hour. (A) Fluorescence emission spectra of 10 μM ThT in the presence of 10 mg/mL native protein (black line) and in the presence of same concentration of amyloid aggregates (red line). (B) Absorbance spectrum of 20 μM Congo red in the presence of 1 mg/mL native protein (black line) and in the presence of equivalent concentration of amyloid aggregates.

Supplementary data

*Circular Dichroism*


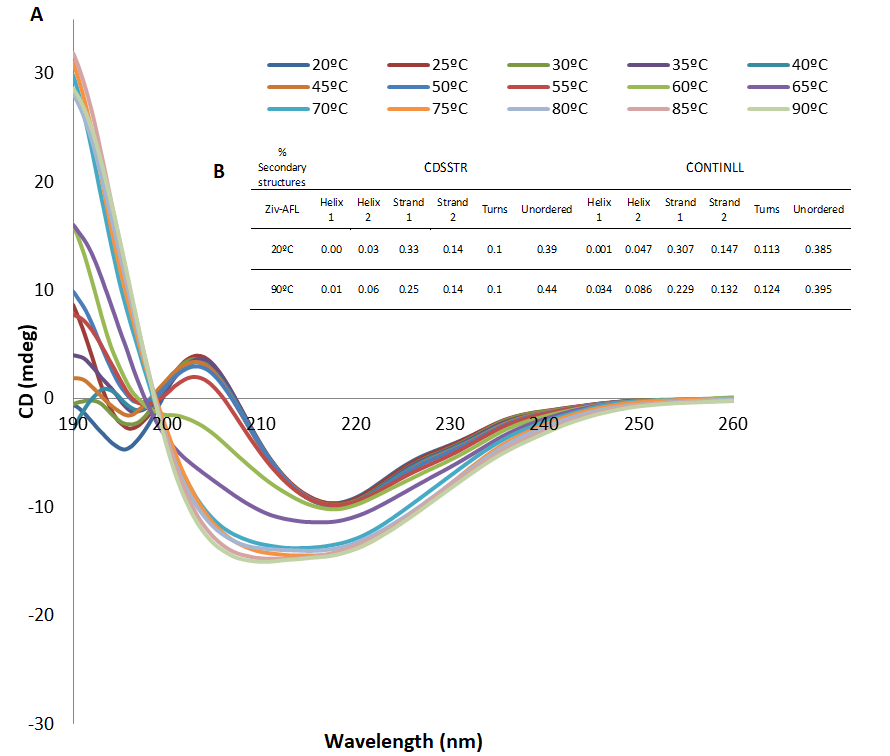


**Figure 2.** (A) Resulting CD spectra of the temperature stability study by submitting ziv-AFL to a temperature ramp (20-90ºC). (B) Secondary structures obtained by Dicroweb^1^ are also displayed.

(1) Whitmore, L. & Wallace, B. A. Protein secondary structure analyses from circular dichroism spectroscopy: Methods and reference databases. Biopolymers 89, 392–400 (2008).

Supplementary data

*Size exclusion chromatography (SE-HPLC-DAD)*


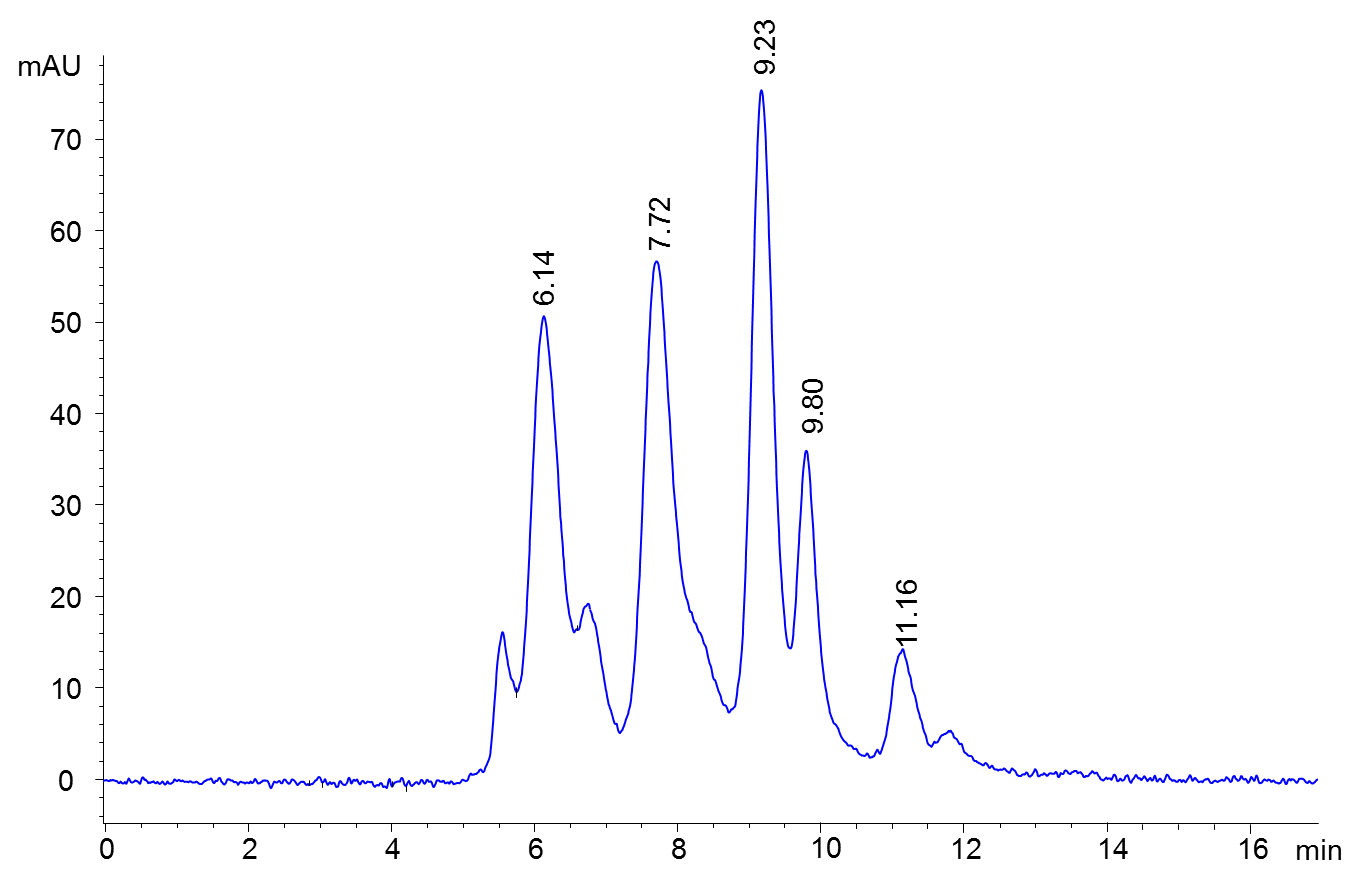


**Figure 3.** Calibration column SE chromatogram. Contains thyroglobulin (6.14±0.02 min), γ-globulin (7.72±0.01 min), ovalmunin (9.23±0.08 min), myoglobin (9.80±0.01 min), angiotensin II (11.16±0.02 min).


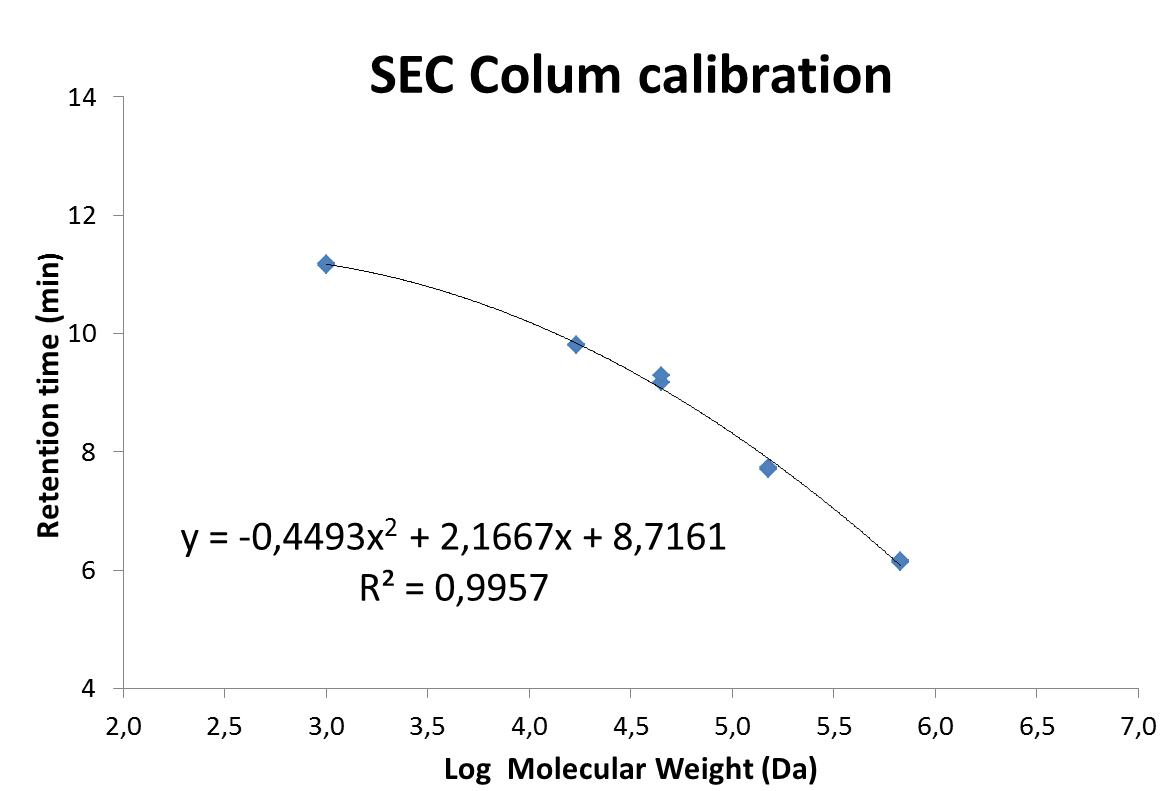


**Figure 4.** Experimental size exclusion column calibration model.

Supplementary data

*Circular Dichroism*

**Table 1.** Percentage estimation of the different secondary structures by Dichroweb^1^ from the CD spectra.

|  | CDSSTR | | | | | | CONTINLL | | | | | |
| --- | --- | --- | --- | --- | --- | --- | --- | --- | --- | --- | --- | --- |
|  | Helix 1 | Helix 2 | Strand 1 | Strand 2 | Turns | Unordered | Helix 1 | Helix 2 | Strand 1 | Strand 2 | Turns | Unordered |
| Control | 0 | 3 | 32 | 13 | 10 | 41 | 0 | 4.6 | 31.2 | 14.7 | 11.6 | 37.8 |
| 60ºC 1h | 0 | 3 | 31 | 13 | 11 | 41 | 0.1 | 4.6 | 30.9 | 14.7 | 11.4 | 38.2 |
| 60ºC 2h | 0 | 3 | 31 | 14 | 10 | 41 | 0.1 | 5.0 | 30.6 | 14.6 | 11.3 | 38.4 |
| 60ºC 3h | 0 | 3 | 32 | 13 | 10 | 41 | 0.1 | 4.8 | 30.9 | 14.8 | 11.3 | 38.1 |
| Light12h | 0 | 2 | 33 | 14 | 11 | 39 | 0 | 4.6 | 31.1 | 14.8 | 11.6 | 37.9 |
| FTC 1 | 0 | 3 | 33 | 14 | 11 | 39 | 0.2 | 4.5 | 30.8 | 14.4 | 11.5 | 38.5 |
| FTC 2 | 0 | 3 | 32 | 14 | 10 | 40 | 0.1 | 4.6 | 31.3 | 14.7 | 11.1 | 38.1 |
| pH 5.2 | 0 | 3 | 33 | 14 | 11 | 39 | 0 | 4.5 | 31.1 | 14.6 | 11.5 | 38.4 |
| pH 7.2 | 0 | 3 | 31 | 14 | 11 | 41 | 0.1 | 4.9 | 31.1 | 14.8 | 11.3 | 37.9 |
| NaCl | 0 | 3 | 34 | 14 | 10 | 39 | 0 | 4.7 | 31.7 | 14.9 | 9.9 | 38.8 |
| GndHcl | 0 | 5 | 29 | 14 | 12 | 40 | 1.3 | 6.1 | 28.3 | 14.1 | 11.6 | 38.7 |

(1) Whitmore, L. & Wallace, B. A. Protein secondary structure analyses from circular dichroism spectroscopy: Methods and reference databases. Biopolymers 89, 392–400 (2008).

1. 🖂 Corresponding author: natalia@ugr.es [↑](#footnote-ref-1)
